# Fibroblast growth factor 20 is protective towards dopaminergic neurons in vivo in a paracrine manner

**DOI:** 10.1016/j.neuropharm.2018.04.017

**Published:** 2018-07-15

**Authors:** Eugene.L. Boshoff, Edward.J.R. Fletcher, Susan Duty

**Affiliations:** King's College London, Institute of Psychiatry, Psychology & Neuroscience, Wolfson Centre for Age-Related Diseases, Guy's Campus, London SE1 1UL, UK

**Keywords:** Fibroblast growth factor 20, Fibroblast growth factor receptor, 6-Hydroxydopamine, Neuroprotection, Parkinson's disease

## Abstract

Neuroprotective strategies are an unmet medical need for Parkinson's disease. Fibroblast growth factor 20 (FGF20) enhances survival of cultured dopaminergic neurons but little is known about its in vivo potential. We set out to examine whether manipulation of the FGF20 system affected nigrostriatal tract integrity in rats, to identify which fibroblast growth factor receptors (FGFRs) might reside on dopaminergic neurons and to discover the source of endogenous FGF20 in the substantia nigra (SN). Male Sprague Dawley rats were subject to a partial 6-OHDA lesion alongside treatment with exogenous FGF20 or an FGFR antagonist. Behavioural readouts and tyrosine-hydroxylase (TH) immunohistochemistry were used to evaluate nigrostriatal tract integrity. Fluorescent immunohistochemistry was used to examine FGFR subtype expression on TH-positive dopamine neurons and FGF20 cellular localisation within the SN. FGF20 (2.5 μg/day) significantly protected TH-positive cells in the SN and terminals in the striatum, while reducing the development of motor asymmetry at 5, 8 and 11 days post lesion. Conversely, the FGFR antagonist PD173074 (2 mg/kg) significantly worsened both the 6-OHDA lesion and resultant motor asymmetry. Within the SN, TH-positive cells expressed FGFR1, 3 and 4 while FGF20 co-localised with GFAP-positive astrocytes. In conclusion, FGF20 protects dopaminergic neurons in vivo, an action likely mediated through activation of FGFRs1, 3 or 4 found on these neurons. Given FGF20 is localised to astrocytes in the adult SN, endogenous FGF20 provides its protection of dopamine neurons through a paracrine action. Boosting the endogenous FGF20 production might offer potential as a future therapeutic strategy in Parkinson's disease.

## Highlights

• FGF20 infusion protects against a partial 6-OHDA nigrostriatal tract lesion in rats.
• Dopaminergic cells in the SNc express FGFR1, 3 and 4 upon which FGF20 can act.
• FGFR blockade exacerbates a partial 6-OHDA nigrostriatal tract lesion in rats.
• Endogenous FGF20 is expressed by astrocytes in the SN, not by dopaminergic neurons.
• Endogenous FGF20 most likely provides protection in a paracrine manner.

## 1 Introduction

Parkinson's disease (PD) is a neurodegenerative condition characterised by both motor and non-motor symptoms. The motor signs largely reflect the progressive loss of dopaminergic neurons in the substantia nigra pars compacta (SNc) and the ensuing striatal dopamine depletion. While current treatments like levodopa or dopamine agonists provide some relief of motor symptoms, there remains no established treatment that protects the dopaminergic neurons, leaving degeneration to progress unchecked. This lack of effective disease-modifying therapy may reflect the multifactorial nature of the underlying pathogenesis of PD which ranges from oxidative stress and excitotoxicity, through mitochondrial dysfunction and neuro-inflammation to genetic predisposition and protein mishandling (AlDakheel et al., 2014). Tackling these pathogenic mechanisms individually might not be sufficient to prevent neurodegeneration. An alternative approach is to increase survival of vulnerable neurons through boosting neurotrophic factors. Given the recent systematic and meta-analysis which concluded that glial-derived neurotrophic factor (GDNF) and neurturin do not improve motor symptoms in people with Parkinson's (Hegarty et al., 2017) it is timely to consider alternative factors that may hold potential.

Fibroblast growth factors (FGFs) have already shown promise in rodent models of PD; for example, FGF2 (Timmer et al., 2007) and FGF20 (Sleeman et al., 2012). Of these, FGF20 has a more restricted distribution (Ernfors et al., 1990; Jeffers et al., 2001) so it presents a more attractive target. FGF20 is selectively expressed in the adult brain, with little to no expression in healthy peripheral tissues (Jeffers et al., 2001; Kirikoshi et al., 2000; Ohmachi et al., 2000). Within the brain, mRNA encoding FGF20 shows further regional selectivity, with highest levels of expression in the cerebellum and substantia nigra (SN) (Jeffers et al., 2001). In vitro, FGF20 protects cultured ventral mesencephalic (VM) dopaminergic neurons against various toxic insults including serum withdrawal, glutamate and 6-hydroxydopamine (6-OHDA) (Murase and McKay, 2006; Ohmachi et al., 2000; Sleeman et al., 2012). Moreover, in vivo, FGF20 infusion protects against loss of dopaminergic neurons in the SNc and against ensuing motor impairments in a rat model of PD bearing a full 6-OHDA-induced lesion of the nigrostriatal tract (Sleeman et al., 2012).

The protective effects of FGF20 in vitro are reduced in the presence of a broad spectrum FGF receptor (FGFR) antagonist or selective MEK1/2 inhibitor (Murase and McKay, 2006; Ohmachi et al., 2003), supporting activation of FGFRs and downstream phosphorylation cascades of the Akt and MAPK pathways playing a key role (Ong et al., 2000; Schlessinger, 2000). mRNA species encoding all FGFRs are found in the brain, with FGFR1, 2 and 3 already reported in SN and striatum (Belluardo et al., 1997; Claus et al., 2004a, 2004b). FGF20 shows affinity for all FGFRs: c isoforms of FGFR1, 2 and 3, and FGFR4 (Ohmachi et al., 2003; Zhang et al., 2006). While the protective effects of FGF20 on dopaminergic neurons are likely to be at least partly mediated through FGFR1, since FGFR1 immunoreactivity is confirmed on dopaminergic TH-positive neurons in rat (Sleeman et al., 2012) and human (Walker et al., 1998) SNc, the involvement of other FGFRs remains to be established.

A role for *endogenous* FGF20 in supporting the survival of dopaminergic neurons is suggested by the findings that prevention of endogenous FGF20 binding to FGFR1c (using the chimeric protein FGFR1c—Fc) led to reduced DA neuron survival in VM mixed cultures (Murase and McKay, 2006). However, whether endogenous FGF20 impacts upon the survival of adult dopaminergic neurons in vivo remains to be determined, as does the nature of any cell types that produce FGF20 in vivo.

The aim of these studies was therefore to further explore the protective role of FGF20 in the nigrostriatal tract in vivo. We hypothesised that exogenous FGF20 would protect against a partial 6-OHDA lesion of the nigrostriatal tract in rats, while treatment with an FGFR antagonist would exacerbate the size of this partial 6-OHDA lesion supporting a protective role for endogenous FGF20. Additionally, we set out to discover which FGFRs were present on dopaminergic neurons in the SNc. Finally, we examined the cellular localisation of FGF20 protein to identify whether dopaminergic neurons or astrocytes were the source of endogenous FGF20 production in the SN.

## 2 Material and methods

### 2.1 Subjects

All studies were carried out in accordance with the UK Animals Scientific Procedures Act (ASPA) and were approved by King's College London animal ethics review panel. A total of 44 adult male Sprague Dawley rats (7-9 weeks; 250-280g; Charles River, Kent UK) were maintained on a 12:12 h light:dark cycle with food and water available *ad libitum*. Of the 44 rats, n = 22 were used for the FGF20 supra-nigral infusion study, n = 19 were used for the FGFR antagonist study and n = 3 were used for localisation of FGF20 and FGFR1-4.

### 2.2 FGF20 supra-nigral infusion in 6-OHDA lesioned rats

Under general anaesthesia (ketamine, 75 mg/kg, i. p.; medetomidine, 0.5 mg/kg, i. p), a dual-barrelled cannula was implanted 2 mm above the right SNc at coordinates AP, +3.7 mm; ML, +2.0 mm; DV, +4.2 mm, relative to the inter-aural line, (Paxinos and Watson, 1993). One barrel was attached via PVC tubing to a pre-filled Alzet 1007D osmotic mini-pump, implanted subcutaneously on the rostral hindback of the rat. Pumps were pre-filled with freshly prepared FGF20 (Peprotech) 83.4 ng/ml or 208 ng/ml in artificial cerebrospinal fluid (aCSF) containing 100 ng/ml of rat serum albumin carrier protein or vehicle (aCSF containing 100 ng/ml rat serum albumin). Pumps provided supra-nigral delivery at 0.5 μl/h supplying treatment groups with vehicle (n = 10), 1 μg/day FGF20 (n = 6) or 2.5 μg/day FGF20 (n = 6) for 1 day prior to and 6 days post 6-OHDA lesion.

After 24h treatment infusion, all rats were subject to a partial 6-OHDA lesion. Rats were pre-treated with desipramine (25 mg/kg i. p.) and pargyline (5 mg/kg i. p.). 30 min later, under isoflurane anaesthesia (5% induction and 2-3% maintenance), 4 μg 6-OHDA in 4 μl 0.02% ascorbate was infused (2 μl/min) via a needle inserted through the second cannula barrel and extending 2 mm further, into the right SNc.

### 2.3 FGFR antagonist treatment in 6-OHDA lesioned rats

Rats were treated once daily with the FGFR antagonist, PD173074 (1 or 2 mg/kg s. c.; n = 6) or PD173074 vehicle (10% DMSO, 10% PEG200 in PBS, pH 7.6; n = 7) for 3 days prior to and 5 days post 6-OHDA lesioning, which was performed exactly as described above.

### 2.4 Measuring motor deficits

Cylinder test assessment of forelimb akinesia was performed in both studies 1 day prior to 6-OHDA lesioning (baseline), and on days 5, 8 and 11 post-lesion (FGF20 study) or days 3 and 5 post lesion (FGFR antagonist study) as previously described (Sleeman et al., 2012). In the FGFR antagonist study, motor asymmetry was additionally measured, 6 days post lesion, using amphetamine-induced rotation test as previously described (Betts et al., 2012). Briefly, animals were injected with amphetamine (5 mg/kg i. p.) and the number of net ipsiversive rotations monitored using rotarat software over a 70-min period.

### 2.5 Tyrosine hydroxylase (TH) immunohistochemistry

Brains were removed on day 12 (FGF20 study) or day 7 (FGFR antagonist study) post 6-OHDA lesioning. Animals were euthanised by overdose with sodium pentobarbitone (Euthatal; 200 mg/ml; 1 ml injection) then intracardially perfused with phosphate-buffered saline followed by 4% paraformaldehyde. Brains were then removed, fixed in 4% paraformaldehyde then embedded in paraffin wax. Serial 8 μm sections of rostral, mid and caudal striatum and SN were obtained and processed for TH staining as previously described (Sleeman et al., 2012).

Digital images of TH-immunostained striatal sections (n = 3 sections per rat each at rostral [AP, +1.6 mm], medial [AP, +0.2 mm] and caudal [AP, -1.4 mm] levels) were acquired and densitometric analysis, corrected for background cortical stain, performed using ImageJ software. TH intensity in lesioned hemisphere was determined as a percentage of intact hemisphere for each animal. Mean percentage TH-immunoreactivity values did not differ between rostro-caudal levels within a treatment group. Data were therefore combined across all three levels, generating a single average value for each animal. Mean data were calculated per treatment group.

Digital images of the nigral sections were acquired at 10× magnification using a Zeiss brightfield microscope. Viable TH-positive cells in the lesioned and intact SNc were counted using ImageJ software. Average number of nigral TH- positive cells per section in the lesioned and intact hemisphere of each animal was taken by averaging the counts from 3 sections at each of the rostral (AP, -4.8 mm), medial (AP, -5.2 mm) and caudal (AP, -5.8 mm) level of the SNc. Data were finally expressed as the number of TH-positive cells remaining in the lesioned SNc as a percentage of the intact side.

### 2.6 Localisation of FGF20 and FGFR1-4 in the rat SN

All immunohistochemistry was performed at room temperature on 8 μm paraffin-embedded sections of the SN from 3 naïve male Sprague Dawley rats (250g; 7-weeks old; Harlan UK). FGF20 and tyrosine hydroxylase (TH)/Glial fibrillary acidic protein (GFAP) co-localisation: Sections were incubated overnight with rat anti-FGF20 antibody (MAB2547, 1:100) alongside either rabbit anti-TH antibody (AB152, 1:1000) or rabbit anti-GFAP (Z0334, 1:500), followed by incubation for 2h with goat anti-rat AlexaFluor 546 (1:200) and goat anti-rabbit AlexaFluor 488 (1:200) solution containing 1 μg/ml Hoechst 33258. FGFRs and TH co-localisation: Sections were incubated overnight with rabbit anti-FGFR1 (F5421, 1:100), rabbit anti-FGFR2 (ab10648, 1:100), mouse anti-FGFR3 (sc-13121, 1:50) or mouse anti-FGFR4 (sc-136988, 1:50) alongside either mouse anti-TH (MAB318, 1:500) or rabbit anti-TH (AB152, 1:500) before incubation for 2h with goat anti-rabbit AlexaFluor 546 (1:200) or goat anti-mouse AlexaFluor 488 (1:200) solution containing 1 μg/ml Hoechst 33258. Fluorescence images were acquired using a Zeiss Apotome fluorescent microscope and Axiovision image analysis software.

### 2.7 Statistical analysis

Statistical analyses were performed using GraphPad Prism 7. Cylinder test asymmetry score was compared between treatments using 2-way Analysis of Variance (ANOVA); differences over time within a treatment group were assessed using 1-way ANOVA with Dunnett's post-hoc test. Total net ipsiversive amphetamine-induced rotations were compared between treatments using a 1-way ANOVA with Dunnett's post-hoc test. Differences in striatal TH-immunoreactivity or TH-positive cell counts in the SNc were compared between treatments using 1-way Analysis of Variance and Tukey's post-hoc test.

## 3 Results

### 3.1 Protective effects of chronic supra-nigral infusion of FGF20 in 6-hydroxydopamine lesioned rats

**Fig. 1:**
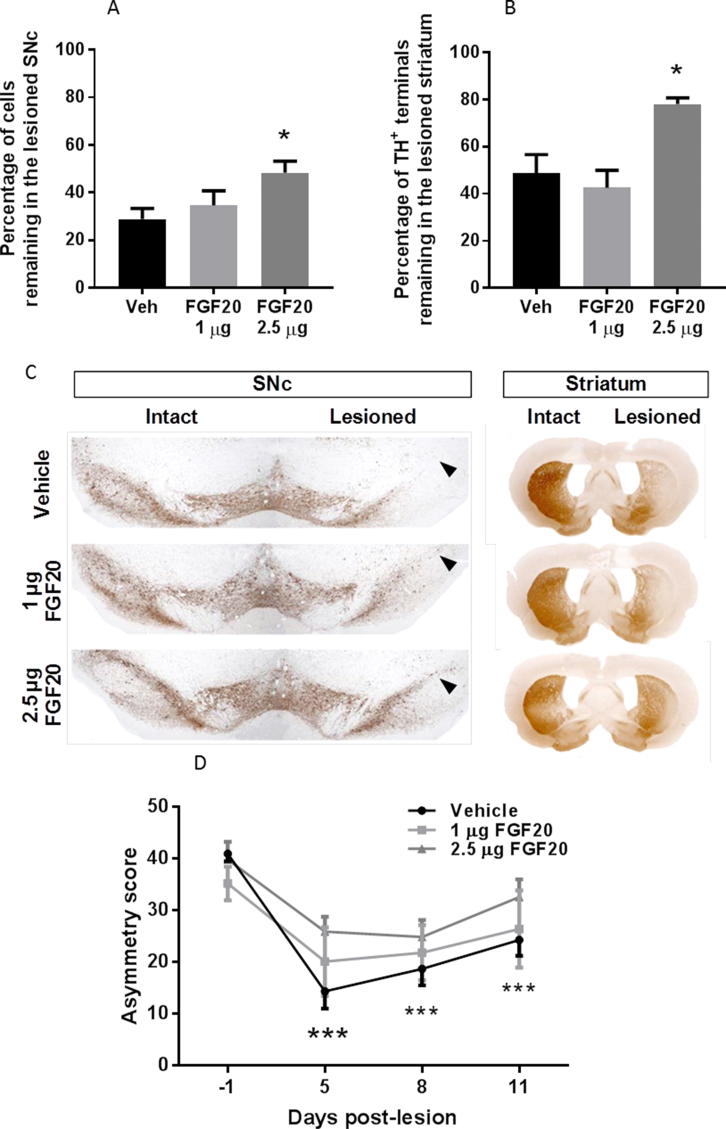
FGF20 protects against dopamine neuron loss and motor deficits in the partial lesion 6-OHDA rat model. Quantification of (A) TH-positive cells in the lesioned SNc and (B) TH immunoreactivity in the lesioned striatum, both expressed as a percentage of the intact hemisphere. (C) Representative images of TH staining in the SNc and striatum. Arrow heads indicate the position of the SNc in the lesioned hemispheres. (D) Quantification of the behavioural asymmetry as measured using cylinder reaching. Data are mean ± SEM, n = 10 (vehicle) or 6 (FGF20 treatment groups); *P < 0.05 compared to vehicle-treated group (A and B); ***P < 0.001 versus pre-lesion score (day -1).

Supra-nigral infusion with the higher dose of FGF20 (2.5 μg/day for 7 days) provided significant protection against the loss of TH-positive cells in the SNc and striatal TH density quantified at 12 days post lesion in the 6-OHDA partial lesion model (P < 0.05; 1-way ANOVA with Tukey's post-hoc test). In the lesion hemisphere of vehicle-treated rats, ∼30% of TH-positive cells remained in the SNc: cell number per section was 105 ± 2 in the intact side, versus 29 ± 5 in the lesion side (mean ± sem; n = 10). In contrast, in the 2.5 μg/day FGF20 infused group ∼50% of TH-positive cells remained in the lesion SNc: cell number per section was 109 ± 3 in the intact side versus 54 ± 6 in lesion (n = 6) (Fig. 1a and b). Similarly, in the lesioned hemisphere of vehicle-treated animals only ∼50% of striatal TH-positive terminals remained, whereas ∼80% remained in the 2.5 μg/day FGF20 group (Fig. 1c and d). FGF20 (1 μg/day for 7d) failed to preserve either TH-positive cells in the SNc or striatal terminals.

Overall analysis of the data revealed a significant effect of time on cylinder test performance (P < 0.05; 2-way ANOVA plus Tukey's post-hoc test). Within-treatment group analyses showed that in vehicle-infused animals, the contralateral forelimb touches were significantly reduced at each time post-lesion compared to pre-lesion (P < 0.001; 1-way ANOVA with Dunnett's post-hoc test). In contrast, there was no significant reduction compared to pre-lesion score for animals treated with either dose of FGF20 (Fig. 1e).

### 3.2 FGFR antagonist infusion exacerbates a partial 6-OHDA lesion of the nigrostriatal tract

**Fig. 2:**
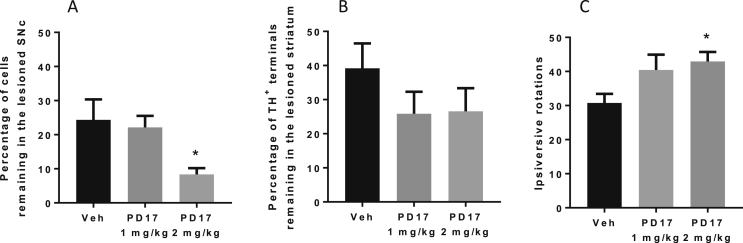
FGFR antagonist, PD173074, exacerbates a partial 6-OHDA lesion in rats. Quantification of (A) TH-positive cells in the lesioned SNc and (B) TH immunoreactivity in the lesioned striatum, both expressed as a percentage of the intact hemisphere. (C) Quantification of the total number of amphetamine-induced rotations. Data are mean ± SEM, n = 7 (vehicle) or 6 (PD173074 treatment groups); *P < 0.05 compared to vehicle-treated group (A and C).

While treatment with FGFR antagonist, PD173074 (1 mg/kg/day for 3 days prior to and 5 days post 6-OHDA) had no impact on the extent of 6-OHDA partial lesion, the higher dose of PD173074 (2 mg/kg/day) enhanced the size of the lesion produced. In vehicle-treated rats, ∼24% of cells remained in the 6-OHDA-lesioned SNc, whereas significantly fewer (8%) remained in the equivalent region of PD173074 (2 mg/kg)-treated rats (P < 0.05; 1-way ANOVA with Tukey's post hoc test). Although not significant, striatal TH loss was also exacerbated following PD173074 treatment (Fig. 2a and b). In the cylinder test, while use of the contralateral paw significantly declined over time as expected post 6-OHDA lesion, there was no worsening apparent with FGFR antagonist treatment (data not shown). However, in the more sensitive amphetamine-induced rotation test, animals treated with the higher dose of PD173074 produced significantly higher total rotations post amphetamine when compared to vehicle-treated animals (P < 0.05; 1-way ANOVA with Dunnett's post hoc test; Fig. 2c).

### 3.3 Localisation of FGF20 and FGFR1-4 in the rat SN

**Fig. 3:**
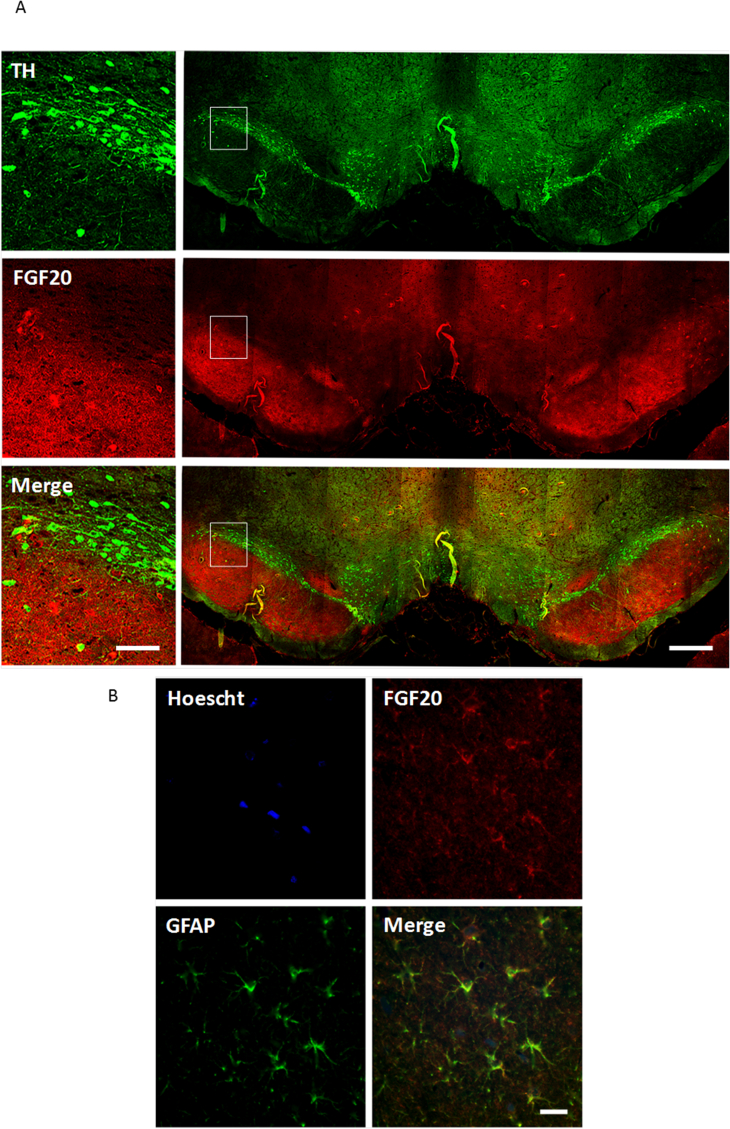
FGF20 co-localises with astrocytes, but not dopamine neurons, in the SN. Immunofluorescent images showing (A) lack of FGF20 (red) signal on TH-positive neurons (green) in the SNc and (B) co-localisation of FGF20 (red) with GFAP (green) in the neighbouring SNr. Images were acquired at:×5 magnification (right hand panel in A; scale bar = 200 μm);×20 magnification (left panel in A; scale bar = 50 μm);×40 magnification (B; scale bar = 20 μm). (For interpretation of the references to colour in this figure legend, the reader is referred to the Web version of this article.)

**Fig. 4:**
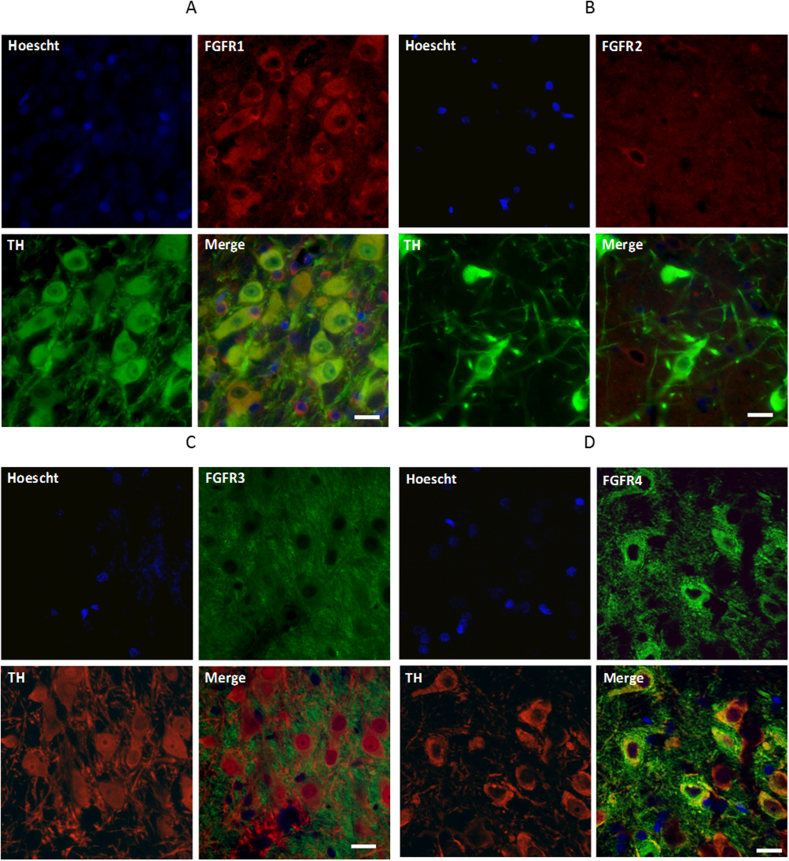
FGFR1, 3 and 4 are expressed on dopaminergic neurons in the SNc. Immunofluorescent images showing co-localisation of (A) FGFR1, (B) FGFR2, (C) FGFR3 and (D) FGFR4 with TH in coronal sections of the adult rat SNc counterstained with the nuclear marker, Hoechst (blue). Intense staining of FGFR1 (red), FGFR3 (green) and FGFR4 (green) is notable on TH-positive stained cells, while FGFR2 (red) shows no co-localisation with TH. All images were acquired at ×40 magnification. Scale bar = 20 μm. (For interpretation of the references to colour in this figure legend, the reader is referred to the Web version of this article.)

The localisation patterns shown are representative of those seen in sections from n = 3 different animals. Immunofluorescent studies in naïve rat brain revealed an absence of FGF20 staining in the SNc and no co-localisation with TH-positive neurons (Fig. 3a). In contrast, FGF20 staining of a diffuse, punctuate nature was observed in the neighbouring SNr where FGF20 was seen to co-localise with the astrocyte marker, GFAP (Fig. 3b). As shown in Fig. 4, FGFR1, 3 and 4 were each expressed on TH-positive dopaminergic cells in the SNc. In contrast, FGFR2 was not expressed on TH-positive cells.

## 4 Discussion

While mounting evidence points to a potential protective role for FGF20 in PD, our understanding of how this might be mediated is far from complete. This study set out to examine the protective role played by FGF20 in the nigrostriatal tract in rats. We report that supra-nigral infusion of FGF20 protects against a partial 6-OHDA lesion of the nigrostriatal tract, an action most likely mediated through activation of FGFR1, 3 or 4 receptors that were shown to co-localise with TH-positive neurons in the SNc. We also found that FGFR blockade exacerbates the extent of a partial 6-OHDA nigrostriatal tract lesion, supportive of a protective role for endogenous FGFs. In the case of FGF20, its absence from TH-positive neurons in the SNc, yet presence in neighbouring GFAP-positive cells suggests a paracrine action whereby FGF20 released from astrocytes activates FGFR1, 3 or 4 on dopaminergic neurons to enhance their survival. These findings reveal potential therapeutic avenues for disease modification in Parkinson's disease.

The protection witnessed here with FGF20 is in line with that we previously reported for a more profound 6-OHDA lesion model of late stage PD (Sleeman et al., 2012) and supports a beneficial role for FGF20 regardless of the severity of the lesion. Given that the in vitro stability of FGF20 at 37 °C (data not shown) was maintained for 4 days before rapidly declining, we conclude that these beneficial in vivo effects occur during the first few days of 6-OHDA lesion when degeneration is occurring, pointing to a protective rather than restorative effect of FGF20. Indeed, using the protocol adopted here, whereby treatment had started by the time the lesion was induced, the focus was on determining a protective effect. Additional studies are required to discover whether FGF20 may also offer benefits when a lesion is already established, for example as a result of sprouting of dopaminergic neurons. Such studies will require extended dosing with FGF20 for between 9 and 12weeks in order to monitor treatment-induced re-innervation without confounding effects of spontaneous re-innervation (Sauer and Oertel, 1994; Kim et al., 2011; Zeng et al., 2012), so alternative delivery mechanisms are required to achieve this.

That FGFR antagonism exacerbates the size of nigrostriatal tract lesion produced by 6-OHDA, supports a role for endogenous FGFs in the survival of dopaminergic neurons. This observation is in line with the aforementioned findings where block of FGFR1 function in vitro, led to a reduced survival of VM dopaminergic cultures (Murase and McKay, 2006). Although neither of these studies can rule out involvement of other FGFs, these data support the notion that endogenous FGF20 has a protective role within the SNc. The source of such FGF20 in the SN is clearly of interest. In their seminal studies, Ohmachi et al. proposed FGF20 mRNA was preferentially expressed in dopaminergic neurons in the SNc (Ohmachi et al., 2000), supporting an autocrine action of this growth factor. However the persistence of FGF20 mRNA in SN homogenates obtained from animals post 6-OHDA lesion of the nigrostriatal tract argued in favour of an alternative source (Grothe et al., 2004). Our findings support the later notion. We found no evidence for FGF20 immunoreactivity in TH-positive dopaminergic neurons in the SNc. Rather, FGF20 was co-localised with GFAP in the neighbouring SNr. We therefore propose that FGF20 released from astrocytes within the SNr diffuses to neighbouring dopaminergic cells in the SNc to provide protection in a paracrine manner.

The presence of FGF20 in astrocytes in the SNr raises the intriguing possibility that, during a 6-OHDA induced lesion with overt inflammatory responses, the infiltration of astrocytes into the area might lead to a compensatory elevation in endogenous FGF20 levels. In animals bearing a partial 6-OHDA lesion, there is no significant elevation in GFAP expression in the SNr (data not shown) so to explore this possibility we utilised previously generated tissue from animals bearing a full 6-OHDA-induced lesion in which a robust inflammatory response is seen. As shown in Supplementary Figure 1, despite a significant elevation in GFAP immunoreactivity in the SNr on the lesion side, there was no significant increase in FGF20 immunoreactivity. Therefore, while resident astrocytes express FGF20, it seems most likely that those infiltrating the region in response to the lesion do not, arguing against any compensatory increase in endogenous FGF20 levels to combat the lesion. Nevertheless, driving enhanced production of FGF20 in the remaining resident astrocytes remains a worthy pursuit in search of a potential protective effect in PD.

The protective effects of FGF20 in vitro are known to be mediated through activation of FGFRs (Murase and McKay, 2006; Ohmachi et al., 2003). To elucidate which FGFRs might mediate the in vivo neuroprotective effects of FGF20, we examined which ones co-localised with TH-positive dopaminergic neurons in the SNc. Consistent with previous reports that FGFR2 was localised purely to astrocytes in the SNc and SNr (Chadashvili and Peterson, 2006; Chadi et al., 2008), we found TH-positive neurons were devoid of FGFR2 expression. The three remaining subtypes (FGFR1, 3 and 4) were all expressed on dopaminergic neurons in the SNc. The presence of FGFR1 corroborates previous findings both in rodent (Sleeman et al., 2012) and human (Walker et al., 1998). Importantly, FGFR1 expression is retained on residual dopaminergic neurons in the SNc from PD brain (Walker et al., 1998) supporting FGFR1 as a potential target for therapeutic intervention. In support of our findings with FGFR3, mRNA encoding FGFR3 was previously noted in the SNc (Belluardo et al., 1997; Claus et al., 2004a), although cellular localisation was not specified. Regarding FGFR4, mRNA encoding this receptor was noted in adult rodent brain (Cool et al., 2002), with a preferential localisation suggested in the medial habenula (Itoh et al., 1994). We likewise observed high levels of FGFR4 immunoreactivity within the medial habenula (data not shown) but also had clear localisation on TH-positive neurons in the SNc. Therefore, activation of FGFR1, 3 or 4 remain possible routes through which FGF20 mediates its neuroprotective effect on dopamine neurons in the 6-OHDA lesioned rat, regardless of whether the source of FGF20 is from astrocytes in the SNr or from exogenous infusion.

In conclusion, these studies have highlighted a protective role for FGF20 in the SNc and identify astrocytes as one potential source of endogenous FGF20. Future work will consider alternative means of delivering FGF20 over a longer duration into the SNc for therapeutic exploration and will identify different approaches to boost the levels of endogenous FGF20 in the brain given the cells producing FGF20 are retained in PD (Tong et al., 2015).

## Funding

This work was supported by a Biotechnology and Biological Sciences Research Council, UK, Centre for Integrative Biomedicine Fund (BB/E527098/1) and a Parkinson's UK project grant (G-1604). The funding sources had no involvement in the study design, conduct, analysis or writing up.

## Conflicts of interest

There are none to report.
