# Supplementary material for: Fibroblast growth factor 20 is protective towards dopaminergic neurons in vivo in a paracrine manner
Source: Neuropharmacology. 2018 Jul 15;137:156–63. doi: 10.1016/j.neuropharm.2018.04.017 (PMC6063078; doi:10.1016/j.neuropharm.2018.04.017)
Supplement: Supplementary Fig [file mmc1.pptx]

## Slide 1
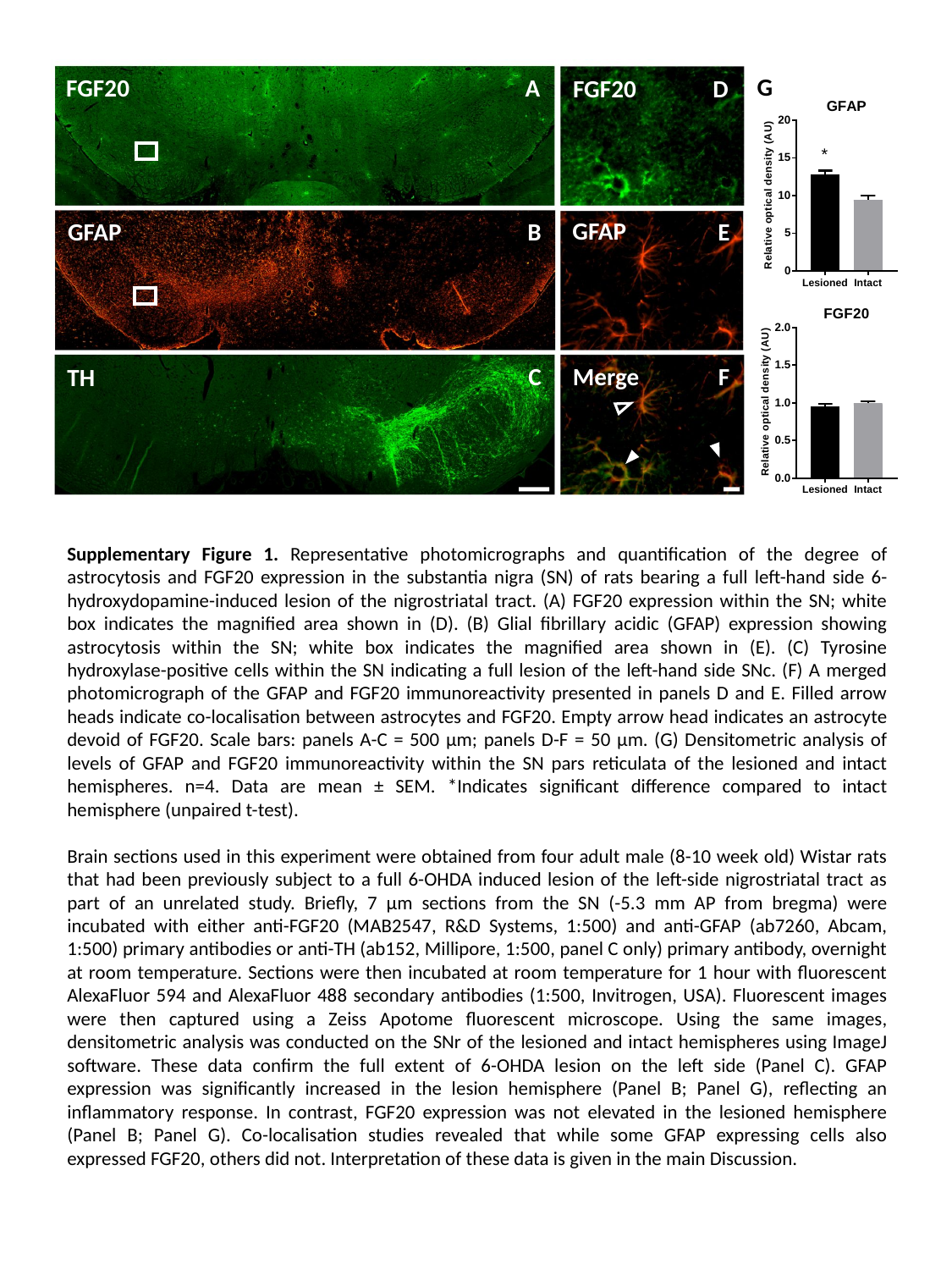

G
FGF20
A
FGF20
E
GFAP
B
GFAP
F
C
Merge
TH
D
E
F
Supplementary Figure 1. Representative photomicrographs and quantification of the degree of astrocytosis and FGF20 expression in the substantia nigra (SN) of rats bearing a full left-hand side 6-hydroxydopamine-induced lesion of the nigrostriatal tract. (A) FGF20 expression within the SN; white box indicates the magnified area shown in (D). (B) Glial fibrillary acidic (GFAP) expression showing astrocytosis within the SN; white box indicates the magnified area shown in (E). (C) Tyrosine hydroxylase-positive cells within the SN indicating a full lesion of the left-hand side SNc. (F) A merged photomicrograph of the GFAP and FGF20 immunoreactivity presented in panels D and E. Filled arrow heads indicate co-localisation between astrocytes and FGF20. Empty arrow head indicates an astrocyte devoid of FGF20. Scale bars: panels A-C = 500 µm; panels D-F = 50 µm. (G) Densitometric analysis of levels of GFAP and FGF20 immunoreactivity within the SN pars reticulata of the lesioned and intact hemispheres. n=4. Data are mean ± SEM. *Indicates significant difference compared to intact hemisphere (unpaired t-test).
Brain sections used in this experiment were obtained from four adult male (8-10 week old) Wistar rats that had been previously subject to a full 6-OHDA induced lesion of the left-side nigrostriatal tract as part of an unrelated study. Briefly, 7 µm sections from the SN (-5.3 mm AP from bregma) were incubated with either anti-FGF20 (MAB2547, R&D Systems, 1:500) and anti-GFAP (ab7260, Abcam, 1:500) primary antibodies or anti-TH (ab152, Millipore, 1:500, panel C only) primary antibody, overnight at room temperature. Sections were then incubated at room temperature for 1 hour with fluorescent AlexaFluor 594 and AlexaFluor 488 secondary antibodies (1:500, Invitrogen, USA). Fluorescent images were then captured using a Zeiss Apotome fluorescent microscope. Using the same images, densitometric analysis was conducted on the SNr of the lesioned and intact hemispheres using ImageJ software. These data confirm the full extent of 6-OHDA lesion on the left side (Panel C). GFAP expression was significantly increased in the lesion hemisphere (Panel B; Panel G), reflecting an inflammatory response. In contrast, FGF20 expression was not elevated in the lesioned hemisphere (Panel B; Panel G). Co-localisation studies revealed that while some GFAP expressing cells also expressed FGF20, others did not. Interpretation of these data is given in the main Discussion.
